# Assessment of Nitrofurantoin as an Experimental Intracanal Medicament in Endodontics

**DOI:** 10.1155/2020/2128473

**Published:** 2020-02-18

**Authors:** Mewan Salahalddin A. Alrahman, Bestoon Muhammed Faraj, Kawa F. Dizaye

**Affiliations:** ^1^Conservative Department, College of Dentistry, University of Sulaimani, Sulaymaniyah, Kurdistan, Iraq; ^2^Medical Pharmacology Department, College of Medicine, Hawler Medical University, Erbil, Kurdistan, Iraq

## Abstract

*Background and Objectives*. Multiple antibacterial agents have been mixed and used as an intracanal medicament-like modified triple antibiotic paste (MTAP) to eliminate *Enterococcus faecalis* (*EF*), which has been most frequently identified in the cases of failed root canal treatment and periapical lesions. This study is aimed at using a single antibacterial agent, nitrofurantoin (Nit), as an experimental intracanal medicament paste against different clinical isolates of *EF*), which has been most frequently identified in the cases of failed root canal treatment and periapical lesions. This study is aimed at using a single antibacterial agent, nitrofurantoin (Nit), as an experimental intracanal medicament paste against different clinical isolates of *Materials and Methods*. Three strains of *EF*), which has been most frequently identified in the cases of failed root canal treatment and periapical lesions. This study is aimed at using a single antibacterial agent, nitrofurantoin (Nit), as an experimental intracanal medicament paste against different clinical isolates of *n* = 90), group M (MTAP) (*n* = 90), group M (MTAP) (*n* = 90), group M (MTAP) (*EF*), which has been most frequently identified in the cases of failed root canal treatment and periapical lesions. This study is aimed at using a single antibacterial agent, nitrofurantoin (Nit), as an experimental intracanal medicament paste against different clinical isolates of *n* = 90), group M (MTAP) (*n* = 90), group M (MTAP) (*n* = 90), group M (MTAP) (*EF*), which has been most frequently identified in the cases of failed root canal treatment and periapical lesions. This study is aimed at using a single antibacterial agent, nitrofurantoin (Nit), as an experimental intracanal medicament paste against different clinical isolates of

**Results:**

Nit could eradicate S1, S2, and S3 completely with concentrations of 6.25, 12.5, and 25 mg/mL, respectively, while MTAP showed complete eradication of the three strains only at 25 mg/mL. In all the groups, it was found that the CFU counts of *EF*), which has been most frequently identified in the cases of failed root canal treatment and periapical lesions. This study is aimed at using a single antibacterial agent, nitrofurantoin (Nit), as an experimental intracanal medicament paste against different clinical isolates of

**Conclusion:**

At the concentration of 25 mg/mL, the Nit paste is effective in eradicating *EF* completely when it is used as an intracanal medicament.*EF*), which has been most frequently identified in the cases of failed root canal treatment and periapical lesions. This study is aimed at using a single antibacterial agent, nitrofurantoin (Nit), as an experimental intracanal medicament paste against different clinical isolates of

## 1. Introduction

Several factors may cause a persistent periradicular infection as a consequence of root canal treatment like intraradicular infection, extraradicular infection, or foreign body reaction and cysts [1]. Those infections are the result of bacterial infection of the root canal, which will end in reinfection and failure of root canal treatment [2]. *Enterococcus faecalis* (*EF*), which is facultative bacteria, is the most predominant and most resistant microorganism leading to persistent periradicular lesions and eventually endodontic failure [3–5]. It is found in root canal failures in nearly 24–70% by culturing methods [6–8] and in 67–77% by molecular methods [9–12]. In other studies, it was retrieved as a major component, about 90% [13, 14]. This microorganism owns many special properties that enable it to survive in root canal and cause reinfection such as the ability to tolerate periods of starvation, deeply invade dentinal tubules [15], antimicrobial resistance, and the ability to adapt to changing environment [16].

Chemomechanical preparation of the root canal is considered the first step to reach the target of eradication of the intracanal bacterial invasion [17]. The chemomechanical procedures can reduce endodontic infection rather than ensure an immaculate root canal system; hence, microorganisms can survive inside the complex anatomy of the root canal system [18, 19]. Therefore, the intracanal medicaments represent an ideal reinforcement step to achieve the complete disinfection of the root canal system [20].

Local application of antibiotics to combat endodontic infections has been an option for years in endodontics, such as intracanal medicaments [21]. It represents a more successful route than systemic antibiotics to prevent the risks of adverse side effects of antibiotics (like allergic reactions or toxicities) [22]. Local application of an antibacterial agent in the form of intracanal medicament gives the chance to target bacteria in each fine locus of the root canal system, which cannot be reached by conventional root canal treatment protocols such as instrumentation and irrigation [23]. Local application of antibiotics like intracanal medicament in endodontics enhances many positive prospects, including a complete or near-complete bacterial elimination, and higher local drug concentrations in addition to minimizing systemic adverse effects [24]. However, this technique may face a problem: the emergence of bacterial resistance [25, 26]. One of the main reasons leading to antibiotic resistance is the incorrect use of antibiotics, which will end in developing resistant microorganisms and, consequently, the formation of resistance genes and their inheritance from antibiotic-resistant to antibiotic-susceptible bacteria [27]. Because of the possibility of high tolerance and antibiotic resistance of *EF* to several antibacterial agents and antibiotics, any antibacterial agent has a short duration of action against *EF* until the development of resistance genes [28–30].

Triple antibiotic paste (TAP), which is a combination of three antibiotics, namely, metronidazole, ciprofloxacin, and minocycline, has been used as an intracanal medicament owing to its high antimicrobial effects [31]. There is a controversy between the studies supporting its efficacy to eradicate *EF* in the root canal system completely [32–35]. This may be due to emerging bacterial resistance [25, 26]. Another drawback of TAP is the crown discoloration due to its minocycline [36, 37]. Therefore, there has been a modification of TAP called modified triple antibiotic paste (MTAP) [38] by replacing minocycline with clindamycin. MTAP was shown to be as effective as TAP in reducing *EF* in the root canal system [39]. Due to those mentioned drawbacks of TAP and its modification to MTAP, there was a need for a new medicament that has less possibility of resistance, is equivalently potent against *EF*, and is preferably a single drug, so it needs less time and effort to prepare, is additionally cost-effective, and is a single drug rather than a multidrug.

Nitrofurantoin (Nit) is a synthetic nitrofuran compound [40]. It is effective against most gram-positive and gram-negative organisms [41]. It is a well-known antibacterial agent widely used as an oral antibiotic treatment for urinary tract infections (UTIs) [42]. Furthermore, it is the drug of choice for the treatment of infections caused by multidrug-resistant pathogens [43–45]. In a study that included 300 isolates of *Enterococcus*, none of the 300 isolates was resistant to Nit, including *EF* [46]. Several studies confirmed that Nit is highly effective against *EF* [47–49].

No previous studies have been done to explore the effect of Nit as a new intracanal medicament within the root canal system against *EF* in endodontics. Therefore, this study assessed the efficacy of Nit paste as an intracanal medicament in extracted teeth in comparison with MTAP.

## 2. Materials and Methods

### 2.1. *Enterococcus faecalis* Strain Source

**Figure 1:**
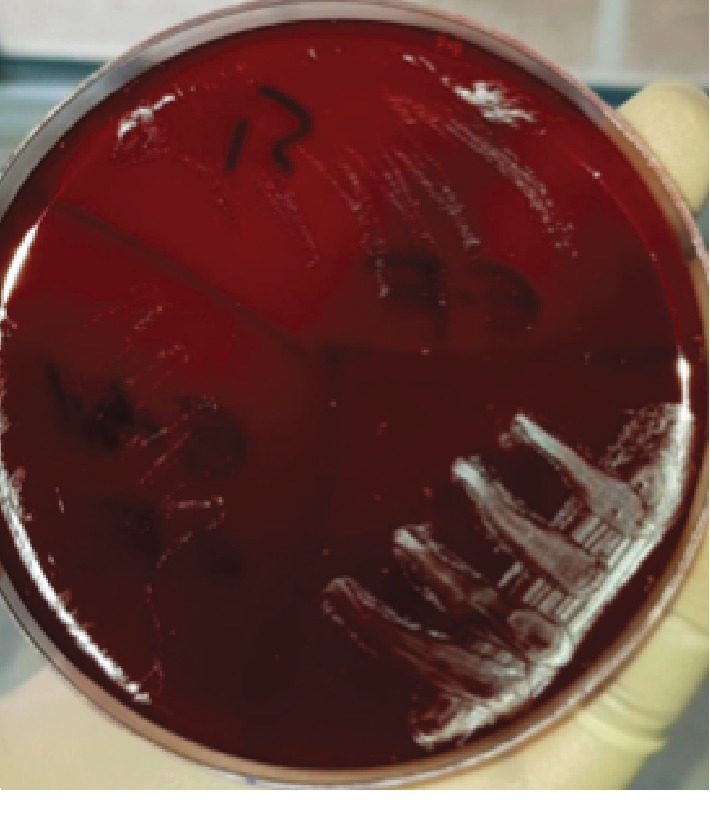
The isolated three strains of *Enterococcus faecalis*.

Three strains of *EF* were used in this study, as shown in Figure 1. Strain 1 (S1) was taken from a blood sample of a sepsis patient, strain 2 (S2) was taken from a failed endodontic patient without antibiotics for the last three months, and strain 3 (S3) was taken from a failed endodontic patient on antibiotics.

### 2.2. Patient Selection

Patients were interviewed and informed thoroughly about the study purpose, and informed written consent was signed before taking the samples of *EF*. The protocol of sampling was approved by the ethical committee at the College of Dentistry, University of Sulaimani.

The first strain S1 was isolated from a patient admitted for sepsis complaining of high fever, rigor, generalized aches and pains, tachycardia, sweating, leukocytosis, elevated ESR (Erythrocyte Sedimentation Rate), and CRP (Compliment Reactive Protein). Blood samples were taken and sent for blood culturing in the bacteriology department, and after 48 hours, *EF* was diagnosed as the causative factor of sepsis in this patient.

The second and third strains S2 and S3 were isolated from two patients complaining of failed endodontic treatment and requiring retreatment.

After obtaining a previous dental history, the patient's chief complaint was documented, and a clinical examination was performed and correlated with radiographic findings.

The two patients selected for the present study were in need of retreatment of their endodontically treated teeth. Each endodontic-treated tooth had a defective coronal seal with incomplete obturation of the root canal that was short filling which was more than 2 mm shorter than the radiographic root apex (radiographic presence of voids and radiolucent space running along some of the working length of the root filling) with a periapical radiolucency demonstrated in the periapical radiograph [50]. Additionally, the two teeth which had been selected for this study could be isolated with a rubber dam with no periodontal pockets more than 4 mm.

The patient from whom the S2 was isolated did not have local or systemic antibiotic administration within the last three months, while the patient who was the source of S3 was on a course of antibiotics for two weeks with no response.

Exclusion criteria to select the two patients with failed endodontic treatment were smoking, pregnancy, diabetes mellitus, autoimmune disease, chemotherapy, immunosuppressive therapy, and malignancy.

### 2.3. Bacterial Sampling

Regarding the first strain (S1), the bacteria were isolated from the blood of the patient and then cultured on blood agar and incubated at 37°C for 48 hours and were examined by a bacteriologist and documented to be *EF*.

The second and third strains (S2 and S3) were isolated according to the procedure of root canal swabbing described by Gomes et al. [7] and Vineet et al. [50]. The selected tooth was isolated with a rubber dam, then it was disinfected with 5.25% sodium hypochlorite (Sultan Healthcare, Pennsylvania, USA); after that, it was inactivated with 5% sodium thiosulfate (The Science Company, Colorado, USA). The whole technique was under aseptic conditions. After removing the tooth filling, the root canal orifice was identified, followed by sterilization of the pulp chamber with 5.25% sodium hypochlorite; previous obturation was removed with Gates Glidden drills (Dentsply, Maillefer, Ballaigues, Switzerland) and endodontic files (Mani, Tochigi, Japan). Sterile saline was introduced inside the canal lumen to wet the canal. Then, two sterile paper points (Dentsply, Maillefer, Switzerland) were inserted into the full length of the canal and kept for 60 s. The paper points were placed into a 3 mL centrifuge tube containing 3 mL of reduced transport fluid (RTF) and transported to the microbiology department to perform the microbiological processing.

### 2.4. Laboratory Assessment

Three different strains were confirmed by the Phoenix and VITEK 2 system (DensiCHEK Plus, bioMérieux, Craponne, France) and by 99% identification with automated sensitivity reporting for all strains. The *EF* strains were cultured in brain heart infusion (BHI) broth (LAB M Limited/Neogen, Lancashire, UK) and incubated at 37°C for 48 h. To achieve a bacterial suspension with a concentration of 0.5 McFarland containing 1.5 × 10^8^ cells/mL, the microbial cells were resuspended with saline [51].

### 2.5. Tooth Preparation

A total of 198 caries-free, straight single-rooted extracted human teeth were collected and stored in 0.9% physiological saline (B. Braun Medical Inc., Pennsylvania, USA) at room temperature until the time of use [52]. (The collection of the extracted teeth was done according to the study protocol approved by the Ethical Committee in the College of Dentistry, University of Sulaimani.) The crowns were cut perpendicularly to the long axis of the teeth from the cementoenamel junction (CEJ), with a rotary diamond disc (15LC diamond wafering blade, Buehler, Illinois, USA) in conjunction with physiological saline irrigation, and kept in 0.9% physiological saline. The root length was cut and standardized to 15 mm. After removing pulp tissue, canals were evaluated for apical patency and checked to have only one canal using #15 K-file (Mani, Tochigi, Japan) (roots with two canals were excluded from the study). The working length (WL) was determined by one mm short of the root apex, using a size 15 K-file, getting a 14 mm WL. The coronal third of the root canal was flared using Gates Glidden drills (#1, 2, and 3) [53], and the canals were instrumented within the WL with the Ni-Ti ProTaper rotary system (Dentsply, Maillefer, Ballaigues, Switzerland) (using sizes of S1, S2, F1, F2, and F3) at 3000 rpm (rotations per minute) speed and 2.5 N cm (Newton centimeter) torque with a micro motor handpiece (NSK-Nakanishi, Tokyo, Japan) (using S1 until 2/3rds of the working length, then Sx was used until the middle third; S1 was used again until the working length, after that using F1, followed by F2 and F3 for full working length) [54].

After each instrument change, 5 mL of 5.25% NaOCl was used for irrigation. Then, the samples were irrigated with 5 mL of 17% EDTA (ethylenediaminetetraacetic acid) (root canal preparation solution, Dline, Estonia, Europe) for smear layer removal. In order to achieve the effects of EDTA, a flush with 5.25% NaOCl for 5 min was done by using a special irrigation syringe. Then, each root was rinsed with 10 mL of physiological saline to remove the remnants of EDTA and NaOCl [55], using an endodontic irrigating syringe (Pacotech Inc., Texas, USA). Finally, the apical foramen of the root was sealed with a bonding agent and light-cured composite resin (Tetric N-Ceram, Ivoclar Vivadent, Liechtenstein) to prevent bacterial leakage. To prevent bacterial leakage from the accessory lateral canals, three layers of clear nail varnish (Orly International Inc., California, USA) were placed over all external root surfaces except for the coronal access and with care not to occlude the root canal entrance, and the teeth were allowed to dry [56].

### 2.6. Sterilization of Specimens

Each root specimen was placed in a sterile test tube containing 10 mL of brain heart infusion (BHI) broth; the tubes were placed in a large laboratory jar and autoclaved twice for 30 min at a temperature of 121°C and a pressure of 15 PSI (Zirbus Technology GmbH, Bad Grund (Harz), Germany) [57]. Then, those samples were kept in an incubator at 37° C for 24 hours (Memmert, Schwabach, Germany). Bacterial viability (contamination) and broth purity were checked.

### 2.7. Inoculation of *Enterococcus faecalis* Bacteria into the Specimens

All the steps of the bacteriology workup were done in a microbiological safety cabinet (Advancelab Pte. Ltd., Senang Cres, Singapore). All the samples (roots) were taken out of the BHI broth test tube aseptically by using a sterile tweezer. Then, the specimen was held by a sterile alcohol pad (70% isopropyl alcohol, Bolikim, China) to prevent contamination of the outer surface of the sample. The broth remaining inside the root canal was removed by aspiration using a sterile disposable syringe with a small gauge needle (BD, Franklin Lakes, New Jersey, USA).

The canals were inoculated with the suspensions of the three different strains of *EF* (S1, S2, and S3), with a standard concentration of 0.5 McFarland (1.5 × 10^8^ CFU/mL), using a DensiCHEK devise (DensiCHEK Plus, bioMérieux, Craponne, France) to measure the optical density for each strain of *EF* suspension. Then, the sterilized canals were filled with 20 *μ*L inoculums of bacteria according to the strains by using a syringe with a sterile endodontic needle without spillage. Then, the orifice of the canal was closed by a sterile small cotton pellet (Kardelen Yazilim, Yenisehir, Turkey) and sealed with a temporary filling (TF) (Dline, Estonia, Europe).

The specimen (root) was wrapped by a wet sterile gauze (Nantong Jianan Medical Products Co. Ltd., Jiangsu, China) and was inserted in a new sterile test tube, and the cap was closed. The tubes were put in a sterile large laboratory jar and incubated for 21 days at 37°C [58].

### 2.8. Sample Grouping

**Figure 2:**
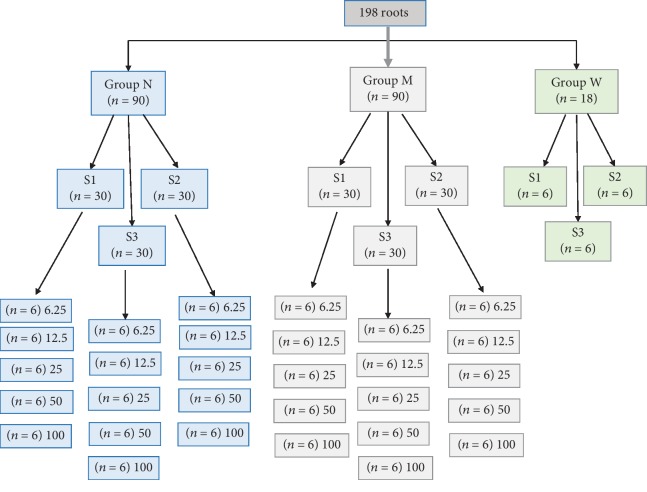
Flowchart showing the distribution of the roots among the groups.

The flowchart of the sample division is described in Figure 2. One hundred ninety-eight roots were divided blindly into three main groups:

- Group N (Nit) (*n* = 90) subdivided into three subgroups (*n* = 30), according to the strain of *EF* (S1, S2, and S3), then each subgroup was divided into 5 groups (*n* = 6) according to the MIC (minimum inhibitory concentration) of Nit used (6.25, 12.5, 25, 50, and100 mg/mL) (as measured in the pilot study)
- Group M (MTAP) (*n* = 90) subdivided into three subgroups (*n* = 30), according to the strain of *EF* (S1, S2, and S3), then each subgroup was divided into 5 groups (*n* = 6) according to the MIC of MTAP used (6.25, 12.5, 25, 50, and 100 mg/mL)
- Group W (*n* = 18) using DW as a negative control, then each group was subdivided into three subgroups (*n* = 6) according to the strain of *EF* used (S1, S2, and S3)

Bacterial viability was checked in three randomly selected tubes for each subgroup.

### 2.9. Preparation of Intracanal Medicaments

Four pure antibacterial powders were used: nitrofurantoin (Nit) (Procter & Gamble Company, Cincinnati, Ohio, USA), ciprofloxacin, metronidazole, and clindamycin (Skywalk Pharmacy, Wauwatosa, Wisconsin, USA).

To calculate the required amount of the antibacterial powder, an analytical balance was used (Sartorius Lab Instruments GmbH & Co. KG, Goettingen, Germany). In this study, we prepared five concentrations of each medicament paste (Nit, MTAP); the concentrations are 6.25, 12.5, 25, 50, and100 mg/mL. To obtain a homogenous antibacterial paste, a magnetic stirrer (Cole-Parmer GmbH, Wertheim, Germany) was used for 2 hours at room temperature.

*Group N (Nit paste)*: Nit solution was prepared by mixing pure powder of Nit with distilled water (DW) (AstraZeneca, Boston, Massachusetts, USA). Methylcellulose (MC) powder (Sigma-Aldrich Chemie GmbH, Schnelldorf, Germany) was added to the Nit solution to get a thick paste-like consistency mixture [59]. 100 mg (Nit) + 1 mL (DW) + 80 mg (MC) were mixed to prepare 100 mg/mL Nit paste, while to prepare 50 mg/mL Nit paste, 50 mg (Nit) + 1 mL (DW) + 80 mg (MC) were mixed, and so on, for the other concentrations.

*Group M (MTAP)*: MTAP was prepared by mixing equal proportions of pure powder of metronidazole, ciprofloxacin, and clindamycin with DW to prepare the MTAP solution. MC powder was added to this MTAP solution to get a thick, paste-like consistency. 100 mg (metronidazole) + 100 mg (ciprofloxacin) + 100 mg (clindamycin) + 1 mL (DW) + 80 mg (MC) were mixed to prepare 100 mg/mL MTAP paste, while to prepare 50 mg/mL MTAP, 50 mg (metronidazole) + 50 mg (ciprofloxacin) + 50 mg (clindamycin) + 1 mL(DW) + 80 mg (MC) were mixed, and so on, for the other concentrations.

*Group W*: 80 mg of MC was added to 1 mL of DW.

### 2.10. Application of the Medicament

After 21 days of incubation, the contaminated roots were taken out of the incubator. Each root was removed from the test tube, and the gauze was unwrapped. The specimen was cleaned with an alcohol pad. The TF and the cotton pellet were removed, and the canal content was aspirated. The aspirated content was cultured on blood agar (Oxoid Limited, Hampshire, UK) for evaluation of bacterial viability and measurement of CFU, then the root canal was irrigated with 5 mL DW to remove the bacterial suspension, and the canal was dried using three paper points. The Nit and MTAP paste were prepared, as mentioned before.

**Figure 3:**
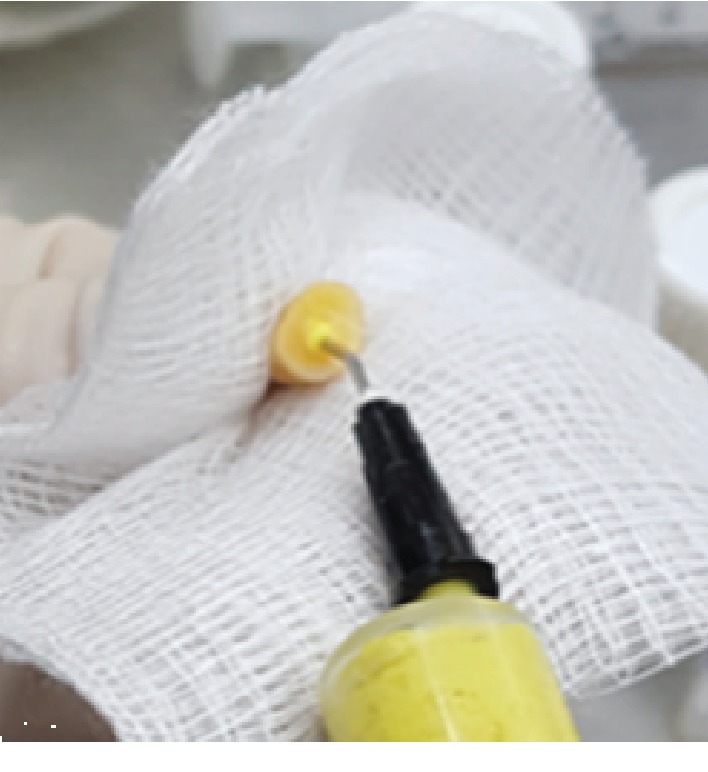
Application of nitrofurantoin paste to the samples.

**Figure 4:**
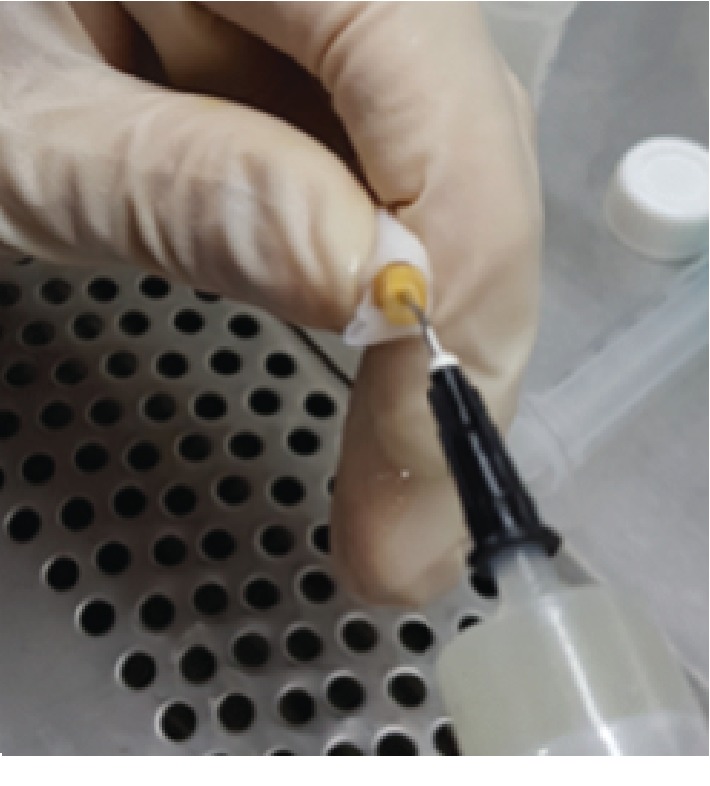
Application of MTAP to the samples.

Each prepared medicament was injected into root canals by using size 27-gauge angled needles until the canal was filled with the medicament paste, as shown in Figures 3 and 4. The roots of the negative control group were injected with DW paste in the same way the medicament was injected. A sterile cotton pellet covered the canal orifice and was sealed with a TF, and the root was wrapped again with sterile wet gauze and placed inside a new sterile test tube. The specimens were returned to the incubator and kept there for seven days at 37°C.

### 2.11. Sampling of the Root Canal Lumen Content

At the end of the seven days of incubation, the specimens were extracted from the test tube, the gauze was unwrapped, the TF and the cotton pellet were removed, and the specimen was held in a sterile alcohol pad. Intracanal medicaments were evacuated from canals by irrigation with 10 mL of DW by using a sterile syringe. Then, two paper points were inserted into the canals and kept for 60 seconds [60]. Then, those paper points were kept in TG (thioglycollate) broth (LAB M Limited/Neogen, Lancashire, UK) through sterile test tubes. They were then incubated at 37°C for 24 hours. Then, subculturing is performed on blood agar at 37°C for 48 h. Growing colonies were counted and recorded as colony-forming units (CFU). To count the colonies of bacteria, we used the classical counting technique in the colony counter, and the results were given as a number of CFU (colony-forming unit).

### 2.12. Sampling of the Dentinal Chips

**Figure 5:**
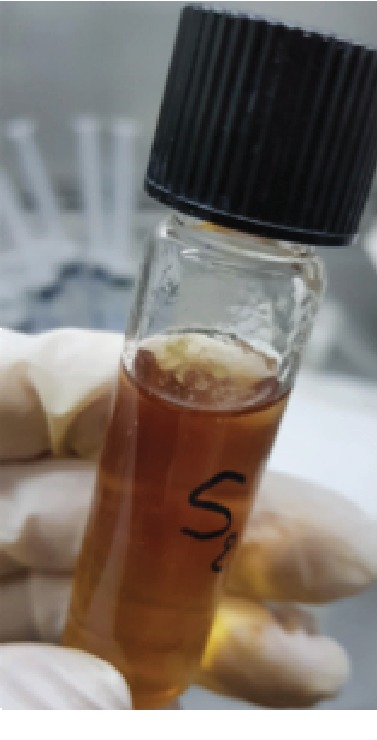
Incubation of dentin chips harvested from the samples.

After the above step, assessment of the extent of infection of the radicular dentin is done depending on dentinal chips, which were obtained by shaving the full length of the root canal using a sterile #40 K-file [61] (tip diameter 0.40 mm) [62]. The dentinal chips were transferred by placing the file (just its cutting surface) into TG (thioglycollate) broth (LAB M Limited/Neogen, Lancashire, UK) through sterile test tubes for 60 seconds (Figure 5). Then, they were incubated at 37°C for 24 hours. Then, subculturing is performed on the blood agar at 37°C for 48 h. Growing colonies were counted and recorded as colony-forming units (CFU).

### 2.13. Statistical Analysis

The results were evaluated statistically by using the Statistical Package for the Social Sciences (SPSS) version 23.0. All the data were expressed as mean ± SD. The Shapiro-Wilk test was used to determine normal distribution of the data. A Student *t*-test was used to compare the results. When the data was not normally distributed, the Mann-Whitney *U* test was used. Changes were considered statistically significant when the *p* value was 0.05 or less.

## 3. Results

### 3.1. Group N

The mean ± SD results of CFU of the three strains of *EF* of this group with different concentrations of Nit and the *p* value comparing CFU of the canal lumen and dentinal chips are shown in (Table 1). In this group, Nit was used at different concentrations (6.25, 12.5, 25, 50, and 100 mg/mL) against the three strains of *EF* (S1, S2, and S3) and the CFU was counted.

*Strain S1*: there was no CFU seen when using Nit at concentrations 6.25, 12.5, 25, 50, and 100 mg/mL.

*Strain S2*: there was no CFU found when using Nit at concentrations 12.5, 25, 50, and 100 mg/mL. When using Nit at a concentration of 6.25 mg/mL, CFU was found, and the CFU of *EF* from the canal (283.33 ± 47.72) was less than CFU of *EF* in dentinal chips (433.33 ± 33.33), and the difference was statistically significant (*p* = 0.028).

*Strain S3*: there was no CFU found when using Nit at concentrations 25, 50, and 100 mg/mL. When using Nit at a concentration of 6.25 mg/mL, CFU was noted, and the CFU of *EF* from the canal lumen (366.66 ± 49.44) was less than the CFU of *EF* in dentinal chips (516.66 ± 47.72), and the difference was statistically significant (*p* = 0.05). On the other hand, using Nit at a concentration of 12.5 mg/mL, CFU was seen, and the CFU of *EF* in the canal (266.66 ± 42.16) was less than CFU in dentinal chips (400.00 ± 63.24), and the difference was statistically not significant (*p* = 0.11).

### 3.2. Group M

The mean ± SD results of CFU of the three strains of *EF* of this group with different concentrations of MTAP and the *p* value comparing the CFU of the canal lumen and dentinal chips are shown in Table 2. In this group, MTAP was used at different concentrations (6.25, 12.5, 25, 50, and 100 mg/mL) against the three strains of *EF* (S1, S2, and S3) and the CFU was counted.

*Strain S1*: there was no CFU when using MTAP at concentrations 25, 50, and 100 mg/mL. When using MTAP at a lower concentration of 6.25 mg/mL, the CFU was recorded and the result of the CFU of *EF* in the canal lumen (183.33 ± 30.73) was less than the CFU of *EF* in dentinal chips (300.0 ± 25.81), and the difference was statistically significant (*p* = 0.016). Also using MTAP at a concentration of 12.5 mg/mL, the CFU was recorded, and the result of the CFU of *EF* in the canal lumen (66.66 ± 33.33) was less than the CFU of *EF* in dentinal chips (191.33 ± 60.09), and the difference was statistically not significant (*p* = 0.120).

*Strain S2*: there was no CFU when using MTAP at concentrations 25, 50, and 100 mg/mL. When using MTAP at a concentration of 6.25 mg/mL, the CFU was recorded and the result of the CFU of *EF* in the canal lumen (271.47 ± 54.26) was less than the CFU of *EF* in dentinal chips (416.66 ± 47.72), and the difference was statistically not significant (*p* = 0.095). Also using MTAP at a concentration of 12.5 mg/mL, the CFU was recorded, and the result of the CFU of *EF* in the canal lumen (200.00 ± 51.63) was less than the CFU of *EF* in dentinal chips (250.00 ± 56.27), and the difference was statistically not significant (*p* = 0.52).

*Strain S3*: there was no CFU when using MTAP at concentrations 25, 50, and 100 mg/mL. When using MTAP at a concentration of 6.25 mg/mL, the CFU was recorded and the result of the CFU of *EF* in the canal lumen (533.33 ± 42.16) was less than the CFU of *EF* in dentinal chips (683.33 ± 60.09), and the difference was statistically not significant (*p* = 0.068). Also using MTAP at a concentration of 12.5 mg/mL, the CFU was recorded, and the result of the CFU of *EF* in the canal lumen (333.33 ± 175.11) was less than the CFU of *EF* in dentinal chips (500.00 ± 57.73), and the difference was statistically not significant (*p* = 0.1).

### 3.3. Group W

The CFU was counted when using DW as a negative control. CFU was seen in all the samples of the three strains of *EF* (S1, S2, and S3). Likewise, the resulting CFU in the canal lumen was less than the CFU in dentinal chips, and the difference was statistically significant between them for the three strains, as shown in (Table 3).

*Strain S1*: there was CFU when using DW, and the result of the CFU of *EF* in the canal lumen (27000.0000 ± 3510.74381) was less than the CFU of *EF* in dentinal chips (39500.0000 ± 4847.3785), and the difference was statistically significant (*p* = 0.01).

*Strain S2*: there was CFU when using DW, and the result of the CFU of *EF* in the canal lumen (28283.3333 + 8584.80893) was less than the CFU of *EF* in dentinal chips (46500.0000 ± 5875.08865), and the difference was statistically significant (*p* = 0.013).

*Strain S3*: there was CFU when using DW, and the result of the CFU of *EF* in the canal lumen (57741.9165 + 4885.35226) was less than the CFU of *EF* in dentinal chips (64333.3333 + 6468.72819), and the difference was statistically significant (*p* = 0.02).

## 4. Discussion

The leading cause of endodontic treatment failure is the persistence of microbial invasion of the root canal system and periradicular tissue [63]. The infection of the root canal system is polymicrobial, containing both anaerobic and aerobic bacteria [64]. The treatment of a root canal is a procedure involving many steps like irrigation and mechanical instrumentation, which is aimed at making the root canals free of bacteria up to 50–70% [65, 66]. So the 30-50% of the root canal which are not bacteria-free will end in intracanal infection and, consequently, periapical infection, leading to root canal treatment failure. That is why intracanal medicaments represent an additional step to achieve complete bacterial eradication, especially *EF* [67, 68].

*Enterococcus faecalis*, which is anaerobic, facultative, and gram-positive bacteria, is considered the most dominant causative microorganism resulting in persistent or secondary infection of root canals, as documented by culturing and molecular methods, leading eventually to failed root canal treatment. *EF* isolated from root canal failure cases owns several factors responsible for the high pathogenesis and persistence inside the root canal system [5, 16, 69]. *EF* produces extracellular protease genes, like gelatinase and serine protease (gelE-sprE operon), which facilitate persistence through biofilm formation. Gelatinase will degrade the organic matrix in dentin, which has a significant predisposition in the infection of the root canal system by *EF*. Furthermore, serine protease can break peptide bonds facilitating adherence of *EF* to dentin [70, 71]. Additionally, there are other genes that help the adhesion of *EF* to the dentinal walls. One such a gene is the Enterococcal surface protein (ESP) gene, which accelerates the virulence, and increases colonization in the root canal system by production of biofilm. This biofilm helps *EF* to withstand the bactericidal effect of antimicrobials by reinforcing the bacteria to become 1000 times more resistant microorganisms to antimicrobial agents than the bacteria that cannot produce such biofilm [72, 73]. Meanwhile, collagen adhesion protein (Ace), antigen A (EfaA), and aggregation substance proteins (Agg) are genes increasing the adherence of *EF*. These adhesion factors will increase the colonization and adherence of *EF* to collagen type I and extracellular matrix proteins found inside the dentin. Also, there is a gene called gelE (secretory metalloprotease gelatinase E), which is another factor responsible for biofilm production in *EF*, causing root canal infection failure [74].

Therefore, *EF* has been selected for this study, as it is the primary and most dominant microorganism found in failed root canal treatment. They have importance because of their resistance to multiple antimicrobials [75]. The three strains were taken in order to evaluate more than one strain of *EF* and to have some diversity and also to assess the possibility of antibiotic resistance that may evolve due to different genes in different strains. Although the resistance characteristics differ in essential ways, they can generally be categorized as intrinsic resistance, acquired resistance, and tolerance [76]. Thus, because of the increasing evidence suggestive of resistance of the *EF* to the commonly used intracanal medicaments [35, 77–79], a more significant effort is done to develop materials that can eliminate *EF* from the root canal system completely.

Nit was selected in this study because it has a broad spectrum of antibacterial activity and is both bactericidal and bacteriostatic against microorganisms [80]. Nit is the drug of choice against *EF*, and it has been used for an extended period in urinary tract infections and chronic and recurrent infections caused by *EF* [81]. Furthermore, resistant species are rare [82, 83]. Nitrofurantoin is a unique antibiotic, owning a hydantoin ring with a nitro-substituted furanyl side chain, which will be metabolized by the bacteria to produce reactive compounds which have bactericidal action on the bacteria [84]. Unlike other antibacterial agents, Nit has a unique mechanism of action. Nit will denature bacterial ribosomal proteins after being reduced by bacterial flavoproteins; this phenomenon will be repeated with other bacterial macromolecules. As a consequence, there will be suppression of many essential processes inside the bacteria like aerobic energy metabolism, cell wall synthesis, DNA synthesis, protein synthesis, and synthesis of RNA. Because of this enormous scope of suppression mechanisms, there is a very poor possibility of developing bacterial resistance to Nit. Thus, bacterial resistance to Nit is very rarely seen since its introduction and FDA approval in 1953 until now. It is very scarce to encounter cross-resistance with antibiotics or transferable resistance in bacteria [85].

We have used MTAP (which is a combination of three antibiotics: ciprofloxacin, clindamycin, and metronidazole) as a control group because we aimed to compare an antibiotic agent (Nit), as an experimental intracanal medicament, with another intracanal medicament (based on an antibiotic agent), furthermore, to assess the efficacy of a single agent compared with a multidrug paste, MTAP, which is a modification of TAP by replacing minocycline with clindamycin to prevent crown discoloration. A severe color change occurred after one day of administration of TAP containing minocycline [86, 87]. Algarni et al. [39] demonstrated that MTAP has a similar efficacy as TAP against *EF* strains. Several studies by Mozayeni et al. [32], Ravi [33], and Sabarathinam et al. [35] showed that TAP resulted in better antibacterial efficacy, against *EF* than nonantibiotic-based intracanal medicaments such as chlorohexidine gel and calcium hydroxide, though it could not achieve complete elimination of *EF*. If not completely eradicated during root canal treatment, *EF* will be transformed into a noncultivable state and will survive the chemomechanical steps that are supposed to be bactericidal. Moreover, that bacteria have the capability to revert into a culturable state when there is a suitable environment [88]. That is why it is necessary to find a medicament that can eliminate *EF* completely.

Any antibiotic has a minimum concentration to kill the bacteria and eradicate it completely called MBC (minimum bactericidal concentration). To achieve this critical concentration, an evaluation of the MIC (minimum inhibitory concentration) should be performed. Therefore, in our study, we used five sequential concentrations of Nit and MTAP including 100, 50, 25, 12.5, and 6.25 mg/mL (as obtained from the serial dilution method that was done in the pilot study). Any concentration resulting in a zero CFU was considered as the MBC.

As a result of this study, regarding the evaluation of Nit and MTAP against the first strain (S1), which was isolated from the blood in a patient with sepsis, Nit showed a complete eradication with zero CFU in the canal lumen as well as the dentinal chips, from the lowest concentration (6.25 mg/mL) onwards, while MTAP could not eradicate this strain from the root canal lumen at the lower concentrations, neither in 6.25 nor at 12.5 mg/mL, but it could achieve a complete eradication with zero CFU at 25 mg/mL upwards. This may be explained by the fact that this strain is isolated from blood in a patient with sepsis so it may have no resistance to Nit but has low resistance to MTAP and also possibly due to the lack of many factors that can contribute to the high resistance of S2 and S3 that were isolated from failed endodontic infections. This can be justified by the fact that this strain demonstrated some resistance to MTAP, which needed a high concentration of MTAP to overcome its resistance, in contrast to the Nit which achieved full eradication even with the lowest one.

On the other hand, when we used Nit and MTAP against the second strain of *EF* (S2), which is isolated from a failed endodontic treatment patient without exposure to antibiotics within the last three months, Nit exhibited complete eradication of this strain with zero CFU in the canal lumen as well as in the dentinal chips at 12.5 mg/mL upwards. Meanwhile, at 6.25 mg/mL, it could not eliminate this strain completely with CFU still seen at the given concentration. Concerning MTAP, it could eradicate this strain completely at the same concentration as that for the first stain which is 25 mg/mL, and it can reduce this strain but not to the degree of complete eradication from the root canal lumen at 6.25 upwards.

Pertaining the third strain of *EF*, being isolated from a failed endodontic treatment patient with an antibiotic course for two weeks' duration with no response, Nit achieved total eradication in the canal lumen as well as the dentinal chips at 25 mg/mL and above, whilst CFU was seen at lower concentrations of 6.25 and 12.5 mg/mL. With regard to MTAP, again, 25 mg/mL was the concentration needed to reach a CFU of zero count.

As perceived from these results, MTAP was noted to show complete eradication at the same concentration (25 mg/mL) regardless of the source of the strain. In contrast, Nit could eradicate S1 and S2 with lower concentrations (6.25 and 12.5 mg/mL, respectively), while for S3, it was 25 mg/mL. This could be explained by the fact that MTAP encountered some resistance from *EF* at lower concentrations (6.25 and 12.5 mg/mL); therefore, it needed higher doses to overcome the resistant bacteria. Since MTAP is a combination of three antibiotics, metronidazole, ciprofloxacin, and clindamycin, the possibility of resistance of *EF* to one or more of those antibiotics will interfere with its antibacterial effect. Duh et al. [89] and Singh et al. [90] found that *EF* was resistant to clindamycin. It is known that enterococci are intrinsically resistant to clindamycin, which is mediated by the product of the lsa gene, although the mechanism remains poorly defined [91]. Furthermore, a study by Dubey and Padhy [92] found that 42% of *EF* was constitutively resistant to clindamycin.

On the other hand, Das et al. [93] found that there was a high resistance of *EF* strains (cultured from UTI) to ciprofloxacin and high susceptibility to Nit. Chayakul et al. [94] showed that the most active drugs against *EF* were Nit. In another study, Gaetti-Jardim et al. [95] evaluated the resistance to antibiotics of species of aerobes and facultative anaerobes isolated from the oral cavity; they found that *EF* was resistant to ciprofloxacin. Rams et al. [96] concluded that metronidazole and clindamycin revealed poor in vitro activity against *EF* isolated from human subgingival samples and would likely be ineffective therapeutic agents against these species in periodontal pockets. However, the clinical isolates were generally sensitive to ciprofloxacin (89.4% susceptible, 10.6% intermediate resistant). Moreover, Lee [97] showed that ciprofloxacin is no longer a recommended therapy for *EF* from complicated UTI, as 47% of the 265 isolated *EF* strains were resistant to ciprofloxacin, whereas Akhter et al. [98] found in their study that 76.19% of *EF* was resistant to ciprofloxacin.

Concerning Nit, Zhanel et al. [46] have shown that Nit is active against all isolates of *EF* found in UTI, demonstrating that they were susceptible to Nit. Butt et al. [81] found that, for a period of three years, Nit was an effective antibacterial in vitro agent and can be used for the treatment of enterococcus urinary tract infections, as they showed that one hundred and twenty-seven (88%) isolates of enterococci were susceptible to Nit. Abdulla and Abdulla [47] showed that Nit was effective against *EF* (cultured from UTI) in 97.3%, while ciprofloxacin was effective in only 35.7%. Rahbar et al. [48] found that Nit had the lowest resistance rate compared to other antibiotics like ciprofloxacin against *EF* (cultured from UTI) (97% vs. 33.38%, respectively). Toner et al. [49] found that *EF* had a sensitivity test 100% to Nit. Sorlozano-Puerto et al. [99] demonstrated that for four years, *EF* had a sensitivity to Nit ranging from 95% to 100%.

To our knowledge, no study is available about the use of pure Nit paste as a single intracanal medicament against *EF* inside the root canal system. Besides, we compared the bacterial growth between inside the root canal lumen and in the dentinal chips after application of the intracanal medicaments. In all of the groups, we found that the number of remaining bacteria (CFU) in the dentinal chips was more than the number of the remaining bacteria inside the root canal lumen.

In group N, the difference between the CFU in dentinal chips and the CFU in the canal lumen was statistically significant when using 6.25 mg/mL against S2 and S3, but it was nonsignificant with 12.5 mg/mL against S3. In group M, the difference between the CFU in dentinal chips and the CFU in the canal lumen was statistically significant when using 6.25 mg/mL and nonsignificant with 12.5 mg/mL against S1, while the difference was statistically nonsignificant for both concentrations against S2 and S3. This is justified by the fact that *EF* colonizes the dentinal walls adhering to the mineral part, probably through LTA (lipoteichoic acids), and to the collagen through AS (aggregation substance) and other surface adhesins [100]; moreover, it has the ability to penetrate the dentinal tubules deeply because of their small size, which is enough for the bacteria to efficiently penetrate the tubules and live within them, in addition to the fact that they can tolerate periods of starvation [71, 101]. Furthermore, Portenier et al. [102] demonstrated that the dentin itself can sometimes antagonize the bactericidal activity of the medicament. Thus, higher concentrations of the medicaments in a thick paste-like consistency are needed to combat these inhibitory effects. This can explain why the higher concentrations of those medicaments used in our study (25 mg/mL) could eliminate *EF* in both dentinal chips and inside the canal system.

The limitation in the present study is that we studied the antibacterial effects of Nit only against *EF*, which is the principal constituent of the microorganisms involved in persistent endodontic infections. Also, it was compared with an antibiotic-based medicament and did not involve other nonantibiotic intracanal medicaments like chlorohexidine.

Further studies are needed to assess the efficacy of Nit against other microorganisms found in polymicrobial infections as it is well known to have antibacterial action against both gram-positive and gram-negative bacteria; combining Nit with an antifungal agent to combat the possible *Candida albicans* species in those infections also warrants further study. Furthermore, we recommend further studies comparing Nit effects with other nonantibiotic-based intracanal medicaments against *EF* and other microorganisms found in polymicrobial infections in root canal treatment failure.

## 5. Conclusions

At a concentration of 25 mg/mL, Nit paste is effective in eradicating *EF* completely when it is used as an intracanal medicament.

## Figures and Tables

**Table 1:** The mean ± SD results of CFU of the three strains of *EF* when using Nit in different concentrations. The *p* value comparing CFU of the canal lumen and dentinal chips.

Strain of *EF*	Site of the sample of bacteria	Concentration of Nit (mg/mL)						
		6.25		12.5		25	50	100
			*p* value		*p* value			
S1	Canal lumen	0	—	0	—	0	0	0
	Dentinal chips	0		0		0	0	0

S2	Canal lumen	283.33 ± 47.72	0.028	0	—	0	0	0
	Dentinal chips	433.33 ± 33.33		0		0	0	0

S3	Canal lumen	366.66 ± 49.44	0.05	266.66 ± 42.16	0.11	0	0	0
	Dentinal chips	516.66 ± 47.72		400.00 ± 63.24		0	0	0

**Table 2:** The mean ± SD results of CFU of the three strains of *EF* when using MTAP in different concentrations. The *p* value comparing CFU of the canal lumen and dentinal chips.

Strain of *EF*	Site of sample of bacteria	Concentration of MTAP (mg/mL)						
		6.25	*p* value	12.5	*p* value	25	50	100
S1	Canal lumen	183.33 ± 30.73	0.016	66.66 ± 33.33	0.120	0	0	0
	Dentinal chips	300.0 ± 25.81		191.33 ± 60.09		0	0	0

S2	Canal lumen	271.47 ± 54.26	0.095	200.00 ± 51.63	0.52	0	0	0
	Dentinal chips	416.66 ± 47.72		250.00 ± 56.27		0	0	0

S3	Canal lumen	533.33 ± 42.16	0.068	333.33 ± 175.11	0.1	0	0	0
	Dentinal chips	683.33 ± 60.09		500.00 ± 57.73		0	0	0

**Table 3:** The mean ± SD results of CFU of the three strains of *EF* when using DW and the *p* value comparing the CFU of the canal lumen and the dentinal chips.

Strain of *EF*	Site of sample of bacteria	Mean ± SD	*p* value
S1	Canal lumen	27000.0000 ± 3510.74381	0.01
	Dentinal chips	39500.0000 ± 4847.3785	

S2	Canal lumen	28283.3333 ± 8584.80893	0.013
	Dentinal chips	46500.0000 ± 5875.08865	

S3	Canal lumen	57741.9165 + 4885.35226	0.02
	Dentinal chips	64333.3333 + 6468.72819	

## Data Availability

The data used to support the findings of this study are included within the article.
